# The Chemical Inhibitors of Endocytosis: From Mechanisms to Potential Clinical Applications

**DOI:** 10.3390/cells12182312

**Published:** 2023-09-19

**Authors:** Olga Klaudia Szewczyk-Roszczenko, Piotr Roszczenko, Anna Shmakova, Nataliya Finiuk, Serhii Holota, Roman Lesyk, Anna Bielawska, Yegor Vassetzky, Krzysztof Bielawski

**Affiliations:** 1 Department of Synthesis and Technology of Drugs, Medical University of Bialystok, Kilinskiego 1, 15-089 Bialystok, Poland; szewczyk.o.k@gmail.com; 2 Department of Biotechnology, Medical University of Bialystok, Kilinskiego 1, 15-089 Bialystok, Poland; roszczenko.piotr@gmail.com (P.R.); anna.bielawska@umb.edu.pl (A.B.); 3 CNRS, UMR 9018, Institut Gustave Roussy, Université Paris-Saclay, 94800 Villejuif, France; anyashm@gmail.com; 4 Department of Regulation of Cell Proliferation and Apoptosis, Institute of Cell Biology of National Academy of Sciences of Ukraine, Drahomanov 14/16, 79005 Lviv, Ukraine; nataliyafiniuk@gmail.com; 5 Department of Pharmaceutical, Organic and Bioorganic Chemistry, Danylo Halytsky Lviv National Medical University, Pekarska 69, 79010 Lviv, Ukraine; holota.serhii@vnu.edu.ua (S.H.); dr_r_lesyk@org.lviv.net (R.L.)

**Keywords:** clathrin-mediated endocytosis, clathrin-independent endocytosis, cell-penetrating peptides, inhibitors of endocytosis

## Abstract

Endocytosis is one of the major ways cells communicate with their environment. This process is frequently hijacked by pathogens. Endocytosis also participates in the oncogenic transformation. Here, we review the approaches to inhibit endocytosis, discuss chemical inhibitors of this process, and discuss potential clinical applications of the endocytosis inhibitors.

## 1. Introduction

**Figure 1:**
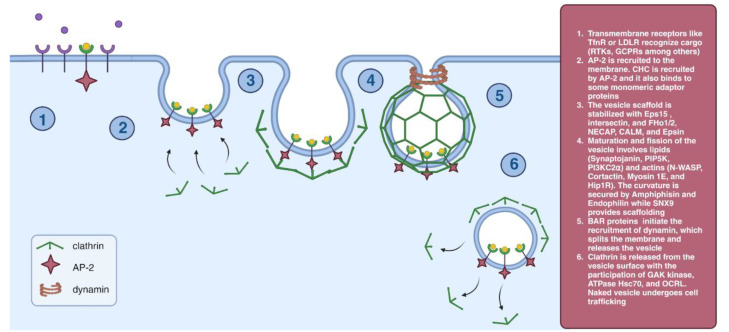
Clathrin-mediated endocytosis.

Endocytosis is a complex process that plays a crucial role in the regulation of numerous intracellular signaling cascades, cell migration, and antigen presentation, among others. Cell sensitivity to certain ligands is modified by the endocytosis of receptors from the membrane surface [1]. Endocytosis results in the formation of membrane vesicles, which transport a variety of cargo molecules from the plasma membrane of eukaryotic cells to the cytoplasm. The cargo consisting of transmembrane proteins and their ligands are involved in a wide range of physiological processes, including cell signaling, nutrient uptake, developmental regulation, and cell adhesion [2]. The most well-recognized endocytosis pathway is clathrin-mediated endocytosis (Figure 1). Clathrin forms a framework for vesicles with cargoes attached to specific receptors; it mediates a large proportion of endocytosis events. However, some cells are capable of clathrin-independent endocytosis [3]. Depending on the involvement of specific proteins or lipids and the ability to internalize specific cargoes, we can divide clathrin-independent endocytosis into several types that we will discuss below [4]. Pathogens can also enter the cell by endocytosis. For infection to occur, viruses must first bind to the outer membrane. This interaction might be nonspecific or occur via viral receptors; it promotes the pathogen entry by initiating conformational changes in the virus itself, activating signaling pathways, and inducing endocytosis [5,6,7]. Endocytosis is also affected in different human pathologies, including cancer, where the deregulation of multiple endocytic proteins and pathways favors metastasis (reviewed in [7]). Endocytosis dysfunction occurs in other pathological conditions, such as heart diseases [8], lipid disorders [9], and atherosclerosis [10], which are undeniably the main contributors to human mortality worldwide. Endocytosis disturbances are also observed in lung diseases characterized by increased contractility, such as asthma and chronic obstructive pulmonary disease (COPD) [11]. Currently, a new therapeutic approach, the delivery of exogenous RNA, has been introduced. Delivery of drugs can be improved by using nanoparticles that enhance endocytosis [12,13]. Endocytic inhibitors were first developed to study this process; currently, these inhibitors are proposed as a potential treatment for some pathologies. Here, we review the basic notions of endocytosis and summarize the data on endocytosis inhibitors and their potential use in clinical practice.

## 2. Mechanisms of Endocytosis

### 2.1. Clathrin-Mediated Endocytosis (CME)

In mammals, the AP-complex family consists of five members, but only adaptor protein complex 1 (AP-1) and AP-2 produce clathrin-coated vesicles (CCV) [14], while the other four AP complexes do not cooperate with clathrin. Beginning with the recognition of short sequence motifs in their cytoplasmic domains, monomeric adaptins and AP-2 bind to the cargo proteins to initiate CME [15]. Some cargo proteins are endocytosed constitutively, while others need to be altered to remove a steric barrier to the binding sequence motif. AP-2 recruits a clathrin heavy chain (CHC), which binds to several monomeric adaptor proteins. The affinity of AP-2 for binding to plasma membranes requires conformational changes in the complex and its subunits. The phosphorylation of its 2-adaptin subunit supports this conformational shift. The conformational change enables cargo protein binding as well as PI-4,5-P2 binding of its α-, β2, and μ2-adaptins. All of these interactions help clathrin attach to the plasma membrane with high affinity and stability [16]. Subsequently, BAR proteins (Bin-Amphiphysin-Rvs) initiate the recruitment of dynamin that forms a helical loop, which, following GTP hydrolysis, splits the membrane and releases the vesicle [17].

### 2.2. Caveolae-Dependent Endocytosis

**Figure 2:**
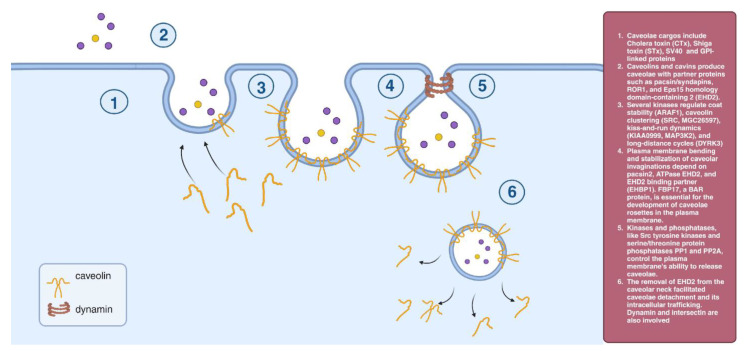
Caveolae-dependent endocytosis.

Caveolin1 (Cav-1) is important for caveolae formation, and approximately 100–200 molecules of Cav-1 are included in a single caveolar coat [18] of 14–16 monomers [19]. Caveolae form in the Golgi complex where Cav-1 oligomerizes and binds cholesterol and fatty acids, which stabilize caveolae formation [20,21]. Cav-1 moves more freely in the plasma membrane, and caveolin flattens due to the decreased cholesterol levels [22]. Plasma membrane bending and stabilization of caveolar invaginations depend on pacsin2, ATPase EHD2, and EHD2 binding partner (EHBP1). The BAR protein FBP17 is necessary for the formation of caveolae rosettes in the plasma membrane. ER/Golgi-level phosphorylation of caveolae membranes might be controlled by the development of Cav-1-dependent domains [23]. Phosphorylation of caveolae membranes at the ER/Golgi level might regulate Cav-1-dependent domain formation. [18]. Caveolae separation and its intracellular trafficking are aided by the removal of EHD2 from the caveolar neck. This process also involves dynamin and intersectin [24,25] (Figure 2).

### 2.3. CLIC/GEEC Endocytosis

**Figure 3:**
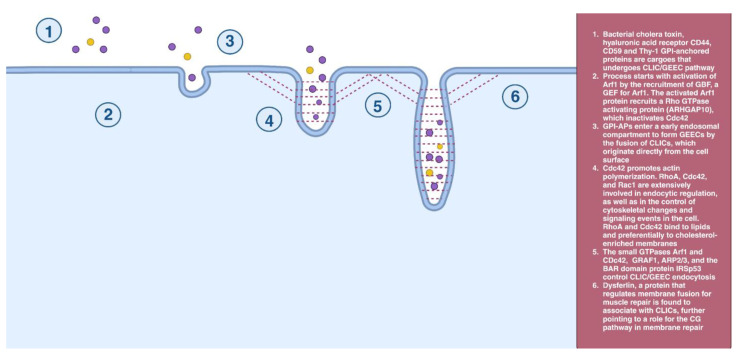
CLIC/GEEC endocytosis.

CLIC (clathrin-independent carrier)/GEEC (GPI-AP enriched early endosomal compartment) endocytosis is clathrin- and dynamin-independent. The glycosylphosphatidylinositol-anchored proteins (GPI-APs) enter a specialized early endosomal compartment through an endocytic pathway independent of dynamin to form GEECs by the fusion of CLICs, which originate directly from the cell surface [26]. The CLIC/GEEC endocytosis has cargoes similar to caveolae-dependent endocytosis (e.g., bacterial cholera toxin, hyaluronic acid receptor CD44, CD59, and Thy-1 GPI-anchored proteins). This pathway requires Cdc42, whose functions include promoting actin polymerization [27]. Members of the Rho family of small G proteins are extensively involved in endocytic regulation, as well as in the control of cytoskeletal changes and signaling events in the cell [28]. RhoA and Cdc42 bind to lipids and preferentially to cholesterol-enriched membranes [29] (Figure 3).

### 2.4. IL2Rβ Pathway 

**Figure 4:**
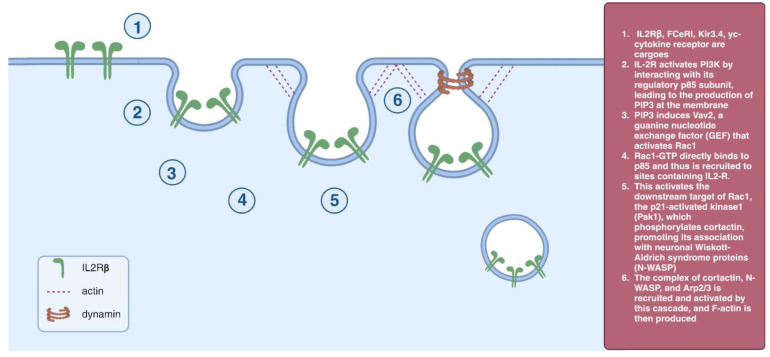
IL2Rβ pathway.

The clathrin-independent pathway responsible for β chain of the interleukin 2 (IL2Rβ) internalization appears to be dynamin-dependent. This process involves small G proteins RhoA and Rac1. Cargo internalizes via small noncoated invaginations. Both GPI-related proteins and the IL2Rβ receptor are enriched in detergent-resistant membrane fractions, while cholesterol withdrawal eliminates the endocytosis of both [30,31]. The GPI-related proteins, IL2Rβ receptor, some flotillin-associated receptors, and amyloid precursor proteins use this route. This process is regulated by an IL-2R-activated PI3P signaling cascade that activates RhoA and Rac1, then p21-activated kinase 1 (Pak1) phosphorylates cortactin to promote its association with N-WASP. The complex of cortactin, N-WASP, and Arp2/3 is recruited and activated by this cascade, and F-actin is then produced [31] (Figure 4).

### 2.5. Arf6-Dependent Endocytosis

**Figure 5:**
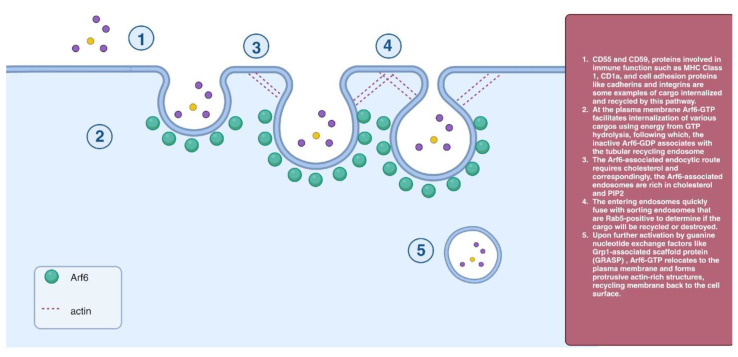
Arf6-dependent endocytosis.

Arf6 is a GTPase found in membranes and endosomal compartments [32]. Arf6 regulates endocytic membrane trafficking and thus affects cell motility, cell division, and lipid homeostasis. Arf6 is also associated with actin remodeling and facilitates ligand internalization at the cell surface, endosomal recycling, and fusion of endosomal and plasma membranes [33]. Arf6 is involved in PIP metabolism, and the effects of its inactivation on AP-2 membrane binding are implicated in a distinct endocytic pathway [34,35]. Actin filaments are required for this process [36], and Arf6-dependent endocytosis is sensitive to cholesterol reduction [37]. The entering endosomes quickly fuse with sorting endosomes that are Rab5-positive to determine if the cargo will be recycled or destroyed. Arf6 overexpression traps cargo in internal vacuolar structures covered with PIP_2_. Thus, the Arf6 inactivation is necessary immediately after internalization for sorting endosomal cargo [38] (Figure 5). 

### 2.6. Flotillin-Dependent Endocytosis

**Figure 6:**
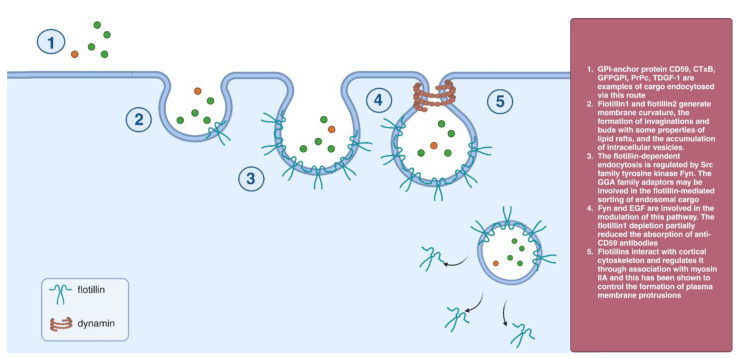
Flotillin-dependent endocytosis.

Flotillin proteins can be found oligomerized in separate membrane domains. Structurally, they possess homology with Cav-1, suggesting that they might organize lipids in a manner similar to caveolae. The flotilla1- and 2-positive domains contain ~95 molecules of each flotillin protein and are morphologically similar to caveolae. [39]. Flotillin1 is required for dynamin-dependent but clathrin- and caveolin-independent uptake of proteoglycans from the cell surface [40]. The flotillin-dependent endocytosis is regulated by Src family tyrosine kinase Fyn. The GGA family adaptors may be involved in the flotillin-mediated sorting of endosomal cargo [41]. Flotillin1 and flotillin2 generate membrane curvature, the formation of invaginations and buds with some properties of lipid rafts, and the accumulation of intracellular vesicles. The number of flotillin1 increases in early endocytic vesicles after fluid-phase uptake of cargo. Fyn and EGF are involved in the modulation of this pathway. The flotillin1 depletion partially reduced the absorption of anti-CD59 antibodies [41,42] (Figure 6).

### 2.7. Phagocytosis

**Figure 7:**
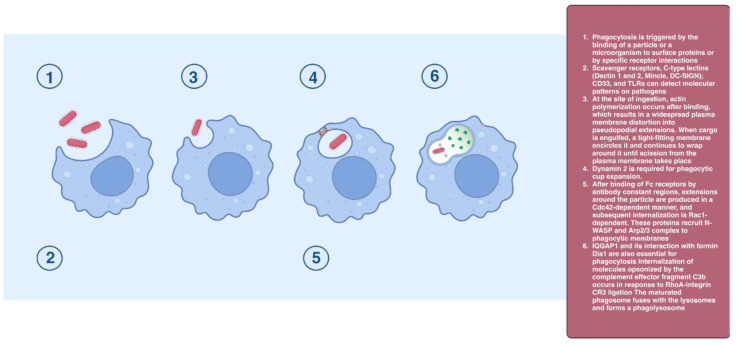
Phagocytosis.

Phagocytosis is triggered by the binding of a particle or a microorganism to surface proteins or by specific receptor interactions. At the site of ingestion, actin polymerization occurs after binding; this results in a widespread plasma membrane deformation into extensions. When cargo is engulfed, a tight-fitting membrane encircles it and continues to wrap around it until scission from the plasma membrane Actin filaments start to depolymerize from the phagocytic cup’s base once the particle has been absorbed; this causes the cup to form a membrane-bound vacuole known as the phagosome. Dynamin 2 is required for the phagocytic cup expansion. The maturation of the phagosome involves the acquisition of Rab GTPases, microtubule-dependent trafficking through dynein/dynactin, recruitment of components of the autophagosomal machinery, and selective retrieval of membrane-associated components. The mature phagosome fuses with the lysosomes and forms a phagolysosome [43,44]. This process depends on small G proteins in the clathrin-independent internalization of opsonized particles. After binding of Fc receptors by antibody constant regions, extensions around the particle are produced in a Cdc42-dependent manner, and subsequent internalization is Rac1-dependent [45] (Figure 7).

### 2.8. Macropinocytosis

**Figure 8:**
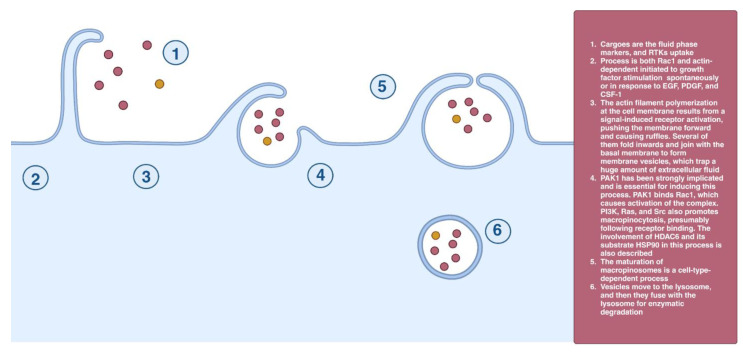
Micropinocytosis.

Macropinocytosis is both a Rac1- and actin-dependent process [46,47]. The actin filament polymerization at the cell membrane results from a signal-induced receptor activation, pushing the membrane forward and causing ruffles. Several ruffles fold inwards and join with the basal membrane to form membrane vesicles, which trap extracellular fluid. They move centripetally in the direction of the lysosome, and then they fuse with the lysosome for enzymatic degradation [46,48]. Serine/threonine-protein kinase (PAK1) is essential for inducing this process [49]. PAK1 binds Rac1, which causes activation of the complex [50]. The activity of phosphatidylinositol-3-kinase (PI3KC3), Ras, and Src also promotes macropinocytosis, presumably following receptor binding. The involvement of histone deacetylase 6 (HDAC6) and its substrate HSP90 in this process is also described [47,51], although the mode of their involvement remains unclear. Macropinocytosis is dependent on cholesterol, which is required for the recruitment of activated Rac1 to these sites [52] (Figure 8).

### 2.9. Fast Endophilin-Mediated Endocytosis (FEME)

**Figure 9:**
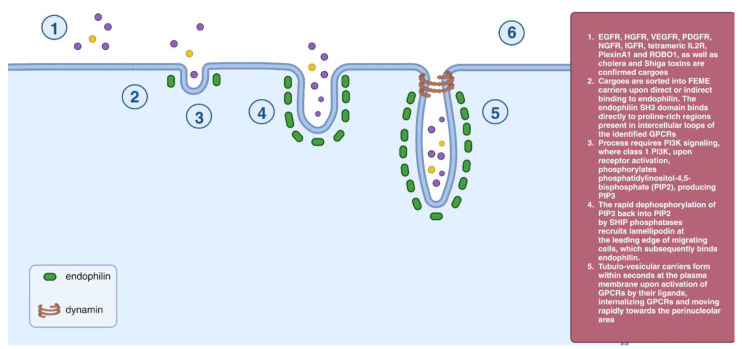
FEME endocytosis.

A clathrin-independent endocytic pathway known as FEME is regulated by endophilin, a BAR domain protein [53]. When particular G-protein-coupled receptors (GPCRs) are activated by their ligands, tubulovesicular carriers rapidly form at the plasma membrane. These carriers internalize GPCRs and move toward the perinucleolar region [54]. PI3KC2/Akt signaling is necessary for FEME as phosphorylation of phosphatidylinositol-4,5-bisphosphate (PIP_2_) is required to produce PIP_3_. Lamellipodin is recruited by PIP_3_ dephosphorylation back into PIP_2_ by the SHIP phosphatases, which then bind endophilin. The SH3 domain of endophilin binds to cargo receptors, the BAR domain causes membrane curvature, and the numerous membrane helices facilitate membrane scission in collaboration with dynamin and actin [55] (Figure 9).

It should be noted that the ratio of clathrin-dependent and clathrin-independent endocytosis can differ depending on the cell type, specific functions, and certain signaling pathways. This leads to differential modulation of intracellular events depending on the signals received [6]. The process itself is not identical in every cell, and its flexibility is represented by the diversity of adaptors and accessory proteins used in the transport of various molecules via the membrane, as discussed above.

## 3. Endocytosis and Pathologies

### 3.1. Cardiac Disorders

Caveolin-dependent endocytosis disruption is essential at the onset of cardiac diseases. The cardiovascular system has a significant expression of Cav-1 and Cav-2. Cav-1, which is extensively expressed in endothelial cells, is necessary for the development and upkeep of caveolae in non-muscle cells. This protein controls vascular development and remodeling, calcium concentration, endothelial nitric oxide synthase (eNOS), and nitric oxide (NO) levels [56].

G-protein-coupled receptors, tyrosine kinases, and signaling enzymes are all caveolin-associated proteins. Consequently, Cav-1 may mediate a variety of cellular consequences [57,58]. Nitric oxide synthase (NOS) plays an important role in the cardiovascular system. eNOS, which catalyzes the conversion of L-arginine to L-citrulline and NO, generates the constitutive vasodilator NO [59,60,61,62].

In contrast, the Cav-1 CSD peptide had no effect on animals lacking eNOS, suggesting that CSD peptides may control vascular disease by way of other proteins [58,63]. Cav-1 peptide injection preserved left ventricular function after reperfusion in isolated rat hearts [64]. Increased NO production and decreased immune cell adhesion were noted, and this was related to the suppression of PKC, a Cav-1-regulated protein that blocks eNOS action [65,66]. Cav-1-deficient animals with hyperactivated ERK 1/2 signaling displayed cardiac hypertrophy [67]. Cav-1-deficient mice also display right ventricular hypertrophy and dilated left ventricles [68].

### 3.2. Lipid Disorders and Atherosclerosis

Lipid disorders and atherosclerosis result from defects in clathrin-dependent endocytosis. Throughout their metabolism, all lipoproteins undergo endocytosis to be degraded intracellularly or to be re-secreted [69]. The identification of low-density lipoproteins (LDL) and the discovery of their transcriptional regulation subsequently led to the development of statins. New factors limiting the intracellular trafficking of LDL and the LDL receptor continue to be discovered as targets for drugs such as Convertase Subtilisin/Kexin Type 9 (PCSK9) [70], IDOL [71], and COMMD/CCDC22/CCDC93 (CCC) [72].

Endocytosis of LDL and residual lipoproteins, as well as the subsequent intracellular accumulation of cholesterol in macrophages, is a key step in the genesis of atherosclerosis [73]. Vascular smooth muscle cells (VSMCs) can migrate from the arterial center with trapped lipoproteins [74,75]. Moreover, VSMCs contribute to as much as 50% of foam cells in intermediate atherosclerotic lesions of coronary arteries. Experiments in mice have revealed that about 30% of all cells in atherosclerotic plaques are derived from VSMCs [76,77,78]. The acquisition of the macrophage phenotype [78,79,80,81] and subsequent transformation into foam cells represents an important early step in the development of the disease. An important stage in the development of atherosclerotic plaque, perhaps even before the formation of monocyte-derived foam cells [82], may be induced by cholesterol loading [83,84] and is probably dependent on lipoprotein endocytosis in vivo. Foam cells express several markers that are also characteristic of macrophage-derived foam cells [76].

### 3.3. Respiratory Diseases

Impaired caveolin-dependent endocytosis may underlie respiratory disorders. Cav-1 immunoreactivity was observed in tracheal and bronchial epithelial cells, smooth muscle, vascular endothelium, airway fibroblasts, and AT1 cells but was absent in AT2 cells and airway epithelial cells in small rat bronchi. Caveolin-2 immunoreactivity showed a similar distribution pattern [85]. Mice lacking caveolin-1 and caveolin-2 have severe lung abnormalities [86,87,88], and abnormal caveolin-1/2 expression is involved in idiopathic pulmonary fibrosis, lung cancer, and pulmonary hypertension [89,90,91].

Obstructive airway diseases, like asthma or COPD, are characterized by airway hyperresponsiveness to inhaled and endogenous bronchoconstrictors [11], accompanied in part by abnormalities in airway smooth muscle spasms [92,93,94,95]. In vitro, caveolin-1 expression increases when airway smooth muscle cells mature to a contractile phenotype [96]. Moreover, the number of caveolae on smooth muscle cells is highest in mature myocytes [97,98]. These observations suggest an important role for caveolin in regulating contractile function.

Caveolins play important roles in mesenchymal cell proliferation. Reducing or silencing Cav-1 expression induces spontaneous proliferation of fibroblasts and smooth muscle cells [96,99,100]. Conversely, overexpression of Cav-1 induces cell cycle arrest and inhibits growth-factor-induced proliferation of smooth muscle cells [101,102,103]. In addition, airway smooth muscle cells and fibroblasts that are in the G0/G1 phase increase endogenous expression of Cav-1 [96,99,100]. Altogether, these data suggest a strong antimitogenic role for caveolin-1 in airway mesenchymal cells, suggesting that abnormal caveolin-1 expression might be involved in fibroproliferative lung diseases.

### 3.4. Cancer

CME, through its effect on signal transduction, is an important player in oncogenesis. This is indicated by the discovery of complex biological mechanisms by which endocytosis may be involved in cell proliferation [104]. Among these is the presence of genetic mutations that involve endocytic proteins in leukemia [105]. The final effect of endocytosis is the posttranslational ubiquitination of endocytic proteins and receptors from the membrane surface as a sorting signal in this pathway [106]. During ubiquitination, a small peptide called ubiquitin is attached to selected proteins by ubiquitin ligases or E3 enzymes. When the ubiquitin in the substrate forms the appropriate chain length, the protein is targeted for proteasomal degradation [107]. However, a single ubiquitin molecule attached to the substrate can perform a signaling function by interaction with ubiquitin-binding domains [106]. Receptor tyrosine kinases, such as the EGFR, have single ubiquitin molecules at multiple sites, which is sufficient for endocytosis and receptor degradation to occur [108]. RTK becomes monoubiquitinated using the E3 ligase Cbl, which acts as an adaptor protein. Interestingly, Cbl transformation and modulation of endocytosis induced by this protein might be involved in oncogenesis [109].

The connection between tumor progression and clathrin-mediated endocytosis was demonstrated by Bao and Yarden, who revealed that active Src, which is a non-receptor tyrosine kinase [110], promotes Cbl destruction. Proteasomal degradation of Cbl was promoted through its tyrosine phosphorylation and polyubiquitination, resulting in increased EGFR expression. Increased Src activity and, consequently, increased surface expression and EGFR signaling occur in tumors [111].

Inhibition of endocytosis is a new promising enhancement of cancer immunotherapy. The antitumor/antipsychotic drug prochlorperazine reversibly inhibits in vivo endocytosis of membrane proteins targeted by therapeutic monoclonal antibodies cetuximab, trastuzumab, and avelumab. Temporary inhibition of endocytosis results in an increased target availability and enhanced efficiency of antibody-dependent cellular cytotoxicity (ADCC) [112] (Table 1).

## 4. Endocytosis of Viruses

Viruses’ small size, simplicity of structure, and absence of self-sustaining metabolic activity limit their active entry into host cells. However, they can promote membrane passage using endogenous cellular responses. At the molecular level, activation of endogenous cellular responses helps viruses to cross membranes and other barriers and deliver their genes to the cytosol or the nucleus. To initiate the entry, viruses first need to bind to the surface of the host cell. This can occur non-specifically through various adhesion factors (heparan sulfate or other carbohydrate structures) [120]. The use of receptors that actively promote entry is an alternative pathway. This can be accomplished by altering the viral particle’s shape through signaling pathways, encouraging endocytosis, or both. Receptors are essential in defining which cell types can be infected because their interactions with viruses are quite selective [5]. Viruses are able to utilize more than one kind of receptor; e.g., HIV-1 uses both CD4 and chemokine receptors [121]. Interestingly, HIV-1 can also bind to heparan sulfate, which may promote the initial recruitment of the virus onto vulnerable cells [120,122].

CME is the most common pathway used by viruses. It transports incoming viruses along with their receptors to early and late endosomes. This process is usually rapid and efficient [123]. Most viruses enter through coated pits, which accumulate under the membrane-bound virus particles. For virus endocytosis to occur via a CME, induction of transmembrane signaling between the virus and the receptor is required [124]. However, it is unclear whether this process is initiated by the recruitment of clathrin coat components to the clustered cytoplasmic domains of viral receptor proteins or by a more complex signaling cascade leading to clathrin recruitment. Entering virions are exposed to the acidic environment of endosomes within minutes after internalization, and changes in pH can lead to viral penetration. However, in some cases, such as Ebola virus and SARS-CoV [125], acidic pH alone is not sufficient to induce fusion, and proteolytic cleavage of viral proteins, particularly via cathepsin L and B, is necessary [126,127,128].

Capsids of SV40 and related polyomaviruses consist of 72 homopentamers, which are similar to the pentamers of the B chain of cholera toxin [129,130]. Both cholera toxin and these polyomaviruses enter cells through caveolar pathways dependent on cholesterol and activation of signaling cascades [131,132,133,134]. Dynamin 2, actin, caveolin-1, and Rho GTPases are involved in the activation of this pathway, depending on the virus and cell type [135]. SV40 internalization by caveolin-dependent endocytosis is regulated by at least five different kinases [136]. Inhibiting them, especially tyrosine kinases, results in a significant reduction in cell infection [135].

A pathway by which a virus can enter into a cell can differ depending on the cell type. Pathways depend on host cell kinases, dynamin, Rac, Rab, and Arf family GTPases, actin and tubulin, and cholesterol [137]. Thus, viruses such as SV40 and influenza can use several different pathways, allowing them to infect a wide range of cells under different conditions.

## 5. Endocytosis of Cell-Penetrating Peptides

The ability to produce cell-penetrating peptides (CPPs) is shared by a number of viruses. Proteins, peptides, siRNAs, and plasmid DNA can all be successfully delivered into cells using CPPs [138]. Typically, these peptides include 5 to 30 amino acids [139].

Depending on the physicochemical characteristics of both the CPP and its cargo, different pathways can be used for CPP uptake [138]. Most of the time, endocytosis takes place in physiological settings and at low peptide concentrations. CPPs can directly cross the cellular membrane at greater concentrations [140]. Full-size and unconjugated HIV-1 Tat peptide, oligo-arginines, as well as anionic CPPs (i.e., MPGα/siRNA complexes, NickFect1 stearylated transportan 10 (TP10) analog), use CME [138]. Caveolae-mediated endocytosis is used by Tat fusion proteins [141], proline-rich CPPs [142], stearylated transportan analogs [143], amphipathic azurin fragments p18 and p28 [138], and N-terminus of VP1 from chicken anemia virus (CVP1) [144]. Octa-arginine (R8) and Tat provoke actin rearrangement in the initial moments of interaction with cell membranes. Macropinocytosis participates in the transport of arginine-rich CPPs such as R8 [145], nona-arginine (R9), dodeca-arginine (R12), Tat peptides and Flock House Virus-derived peptide [146,147,148,149].

Constitutive synthesis of HIV regulatory proteins in infected brain cells may lead to neurological disorders since combinational antiretroviral treatment (cART) does not suppress the expression of HIV non-structural proteins. Despite the fact that HIV-1 replication is efficiently regulated, people with HIV-1 experience chronic inflammation, which suggests that processes other than viral replication are to blame for these individuals’ neurological problems. [150]. Chronic low-level Tat production has been associated with ceramide accumulation, synaptic and axonal degeneration, astrocyte activation, inflammatory cytokine release, and decreased brain function [151].

## 6. Endocytosis of Nanoparticles

Nanotechnology improves overcoming limitations of conventional compounds, such as the insufficient ability to move across membranes or particle distribution. Nanoparticles (NPs) enhance the stability and solubility of compounds and increase the drug’s residence time in circulation. NPs enter cells by endocytosis and accumulate mainly in lysosomes and mitochondria, impairing their physiological functions. The uptake of nanoparticles into the cell depends on a number of physical parameters [152].

Particle size is clearly one of the critical parameters in endocytosis. Caveolin-based vesicles generally are smaller (average 60 nm) compared to clathrin-based vesicles (average 120 nm); therefore, larger NPs are taken up by cells via the clathrin-dependent pathway. Particles larger than 4500 nm enter cells only by phagocytosis or macropinocytosis, while other endocytosis mechanisms are limited in cargo size, with a maximum of ~200–300 nm [153].

Nanoparticle shape also affects its uptake by the cell. Differences in cell membrane curvature, reduction of available receptor binding sites, uneven protein coverage, and lack of multivalent binding to receptors are involved [154].

The surface charge of NPs affects their behavior in biological environments. The surface charge of NPs may depend on biomolecules adsorbed on the surface or on the pH of the environment. The internalization of cationic NPs is more efficient compared to neutral and anionic NPs [155,156]. Further information on the endocytosis of nanoparticles can be found elsewhere [157,158].

## 7. Endocytosis Inhibitors: Mode of Action

To better understand different types of endocytosis, scientists have looked for ways to block this process using non-specific and specific chemical inhibitors as well as genetically engineered cells or organisms carrying endocytic genes or protein knockouts.

### 7.1. Non-Specific Endocytosis Inhibitors

#### 7.1.1. Potassium Depletion

Endocytosis can be arrested by intracellular K+ depletion. When intracellular K+ levels fall below a threshold of 40% of the physiological values, the surface binding of LDL and EGF is not altered, but the internalization of ligands is severely inhibited. The arrest of endocytosis, in this case, is associated with a significant reduction in the number of coated pits and an apparent decrease in the presence of clathrin on the cell membrane. Subsequent addition of KCl to the culture medium restores intracellular K+ levels and endocytosis and leads to clathrin apparition in CCPs [159].

#### 7.1.2. Hypertonic Medium

Hypertonic medium inhibits receptor-mediated uptake of peptides without affecting macropinocytosis by multinucleated leukocytes. Cells in a hypertonic environment do not accumulate the peptide; however, cells still form and process endosomes containing liquid phase markers. This inhibition is independent of the solvent: sodium chloride, sucrose, and lactose inhibit uptake to a similar degree. The hypertonic medium had little effect on saturated peptide binding; however, it prevented the clustering of surface molecules [160].

#### 7.1.3. Cytosol Acidification

A mutant fibroblast cell line lacking the Na/H+ transporter was used to investigate the effect of low cytoplasmic pH on membrane transport in the endocytic and exocytic pathways. Cells were acidified from pH 6.2 to 6.8 for 20 min. Acidification of the cytoplasm does not affect intracellular ATP levels or the number of clathrin-coated pits on the cell surface. However, acidification of the cytoplasm below pH 6.8 blocks the uptake of fluid phase markers, as well as the internalization and recycling of transferrin. Both macropinocytosis and receptor-induced endocytosis restart when the pH of the cytoplasm returns to physiological values. Low cytoplasmic pH also inhibits the rate of intracellular transport from the Golgi complex to the plasma membrane. Acidification of the cytosol to pH < 6.8 reversibly inhibits membrane transport of vesicular stomatitis virus (VSV) by both endocytic and exocytic pathways. Clathrin- and non-clathrin-coated vesicles, which are involved in endo- and exocytosis, cannot detach from the cell surface below the critical internal pH value [161].

#### 7.1.4. Temperature Decrease

A decrease in temperature is a universal inhibitor that suppresses the process of endocytosis and exocytosis. Using a line of rabbit alveolar macrophages with an inflection point at 20°, the endocytosis of labeled serum albumin and the exocytosis of labeled lysine were examined at various temperatures. Below 10°, no ligand degradation was noticed [162].

### 7.2. Clathrin Inhibition

Clathrin is a protein used in clathrin-dependent endocytosis [163,164], mitosis, or recycling of synaptic vesicles [165,166]. Its activity is determined by the formation of a complex called triskelion, which consists of three heavy chains of clathrin, each linked to a light chain. The three arms of the triskelion are flexible to allow the formation of various diameter vesicles by polymerizing units [167]. Two human clathrin heavy-chain isoforms have been identified: CHC17, which is important in membrane maintenance and mitosis, and CHC22, which is mostly present in skeletal muscle. The amino acid homology between the two isoforms is 85%. The CHC17 form can interact with two light clathrin chains that are 60% homologous to one another [168]. During these interactions, the hydrophobic rests of the light chains turn toward the heavy chains. The C-terminal is located near the apex of the triskelion [169]. The inhibitors reviewed in this section do not have a clearly defined capture point but have proven activity against clathrin.

Six segments make up the heavy chains of clathrin: proximal, knee, distal, ankle, linker, and terminal domain. For a discussion of terminal domain inhibition, which is the main target for developing inhibitors, see Section 7.3.

#### 7.2.1. Bolinaquinone (BLQ)

**Figure 10:**
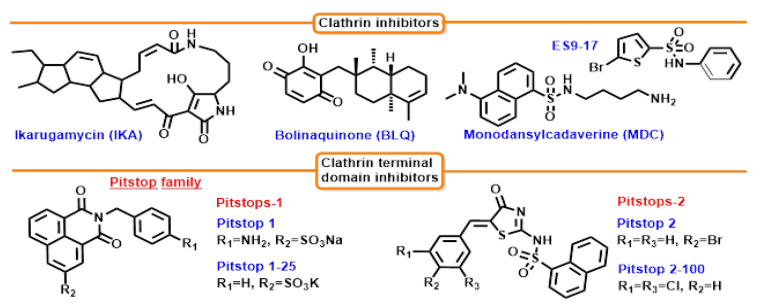
Structures of clathrin inhibitors.

The natural marine hydroxyquinone terpenoid bolinaquinone (BLQ) placed on a carrier, after modification by adding the a,o-diaminopolyethylene glycol chain (Figure 10), inhibits CME. BLQ inhibitory effect on CME was confirmed using microscopy and flow cytometry in direct proportion to the dose [170].

#### 7.2.2. Ikarugamycin (IKA)

*Streptomyces phaeochromogenes* is the source of ikarugamycin. IKA was formerly categorized as an antiprotozoal chemical but is now a widely used antibiotic. IKA prevents the uptake of oxidized low-density lipoproteins and can suppress CME both in plant and mammalian cell lines. The uptake of the transferrin receptor was seen to decrease in an IKA dose-dependent manner. The IC_50_ was 2.7 ± 0.3 μM in the H1299 line preincubated with IKA for 1h. In H1299, HCC366, and ARPE-19 cells, TfnR uptake was decreased by 80%; in H1437 and HBEC3KT cells, 50%. These findings imply that IKA can prevent TfnR uptake. IKA alters the morphology of the CCP, which causes a redistribution of AP-2 and CHC. The exact mechanism is unknown [171].

#### 7.2.3. ES9-17

A mitochondrial uncoupler, endosidin 9 (ES9), was identified as an inhibitor of CHC and what follows CME function in both *Arabidopsis* as well as human cells through in vitro binding studies and X-ray crystallography. A chemically improved analog of ES9, ES9-17, does not have the side effects of ES9 and preserves its ability to target CHC [172].

#### 7.2.4. Monodansylcadaverine (MDC)

Monodansylcadaverine (MDC) is an in vivo marker for autophagic vacuoles and a relatively specific blocker of CME. The inhibitory activity of MDC has been attributed to the stabilization of clathrin-coated pits. However, evidence for this mechanism has only been obtained in cell-free systems using purified clathrin and very high concentrations of MDC. It remains to be investigated whether similar stabilization of clathrin-coated pits can be achieved at lower (100–300 µM) concentrations of MDC, which impair endocytosis in living cells [173].

### 7.3. Clathrin’s Terminal Domain Inhibition

Clathrin terminal domain (CTD) is a central node of protein–protein interactions [6]; overexpression of clathrin-binding endocytic protein fragments results in clathrin uptake within the cytoplasm [174], suggesting that multiple abundant accessory protein interactions involving the CTD serve to recruit clathrin to membranes. Seven sheets are joined to form the propeller-like CTD [175]. Small helical segments are located in the prolonged gap between blades 1–7 and 3–4 of the rotor. In order to connect with adaptor proteins and enable direct cargo attachment, the terminal domain expands inward. Short linear peptide sequences are used by adaptors to bind to the terminal domain [176]. A brief helical linker, followed by an ankle region, connects the CTD to the distal leg [177]. ADP-ribosylation factor-binding protein (GGA1) and the beta chains of AP-1 and AP-2 have sites in the ankle. Important C682 and G710 residues can be found in this binding site [178].

Several more clathrin-binding sites exist. The rest of the essential residues for binding (Q89, F91, K96, and K98) are situated between the blades of the first structure, known as the clathrin box, which corresponds to the propeller’s blades 1 and 2 [175,176]. The W-Box, which binds proteins with the sequence PWXXW, where X is any amino acid, is the second binding site. I154, F27, Q152, and I170 are the important residues at this position [176,179]. Arrestin 2L binding occurs at another position. Indicated by T235, V190, Q192, W164, L183, E232, R188, 231, I194 and K245, it is located between blades 4 and 5. A binding motif is present in the AP-2 -2 subunit, perhaps making it possible to attach to both site 1 and site 3 [180]. The final suggested location has the following residues: N175, G179, R221, F252, Q23, and F260 between blades 5 and 6. A molecular dynamics investigation of putative bolinaquinone binding sites led to the suggestion of this location [181,182].

#### Pitstop Family

Pitstops were designed as inhibitors of the terminal domain of clathrin, binding to the clathrin-box motif site (Figure 10). The effect of rhodanine-based pitstop 2 on clathrin-dependent endocytosis is observed within minutes of cell exposure to the compound. [183]. Treatment of human cultured cells (BEAS-2b, COS-7, HeLa) with Pitstop2 at a concentration of 20 mM resulted in blocking the internalization of transferrin and MHCI, although MHCI still could bind to the cell surface. Endocytosis of CIE-translocated proteins was verified in the presence of pitstop2, CD59 protein (which has the same translocation pathway as MHCI) anchored to GPI was blocked by this substance. Other proteins (CD44, CD98, and CD147) that have a different translocation pathway than CD59 also had their internalization inhibited, and in the control sample, these proteins were observed in recycling endosomes. Inhibition of endocytosis by pitstop 2 occurs after 10 min of exposure and is reversible. In COS-7 and BEAS-2B cells, the same effect was observed on the two types of endocytosis by blocking the n-terminal domain of clathrin. Inhibition of transferrin and MHCI endocytosis was also observed in cells lacking the µ2 subunit of AP2 or the heavy chain, suggesting that pitstop blocks CIE independently of clathrin [184].

### 7.4. Inhibition of Dynamin

The superfamily of dynamin-like proteins, which are GTPase-type proteins, includes dynamin [185]. Both humans and more basic species like bacteria include the existence of dynamin [186]. Dynamins set themselves apart from normal GTPases by having a larger GTPase domain (~300 amino acid residues), a more developed capacity for self-organization [187,188,189], and stronger activity to degrade GTP [190,191]. Dynamin is recruited during the creation of clathrin-coated vesicles to separate them from the extracellular membrane, and it has a size of 100 kDa [192]. Mammals have three different forms of dynamin, known as classical dynamins (I, II, and III) [193,194]. Both share the same domain organization and are 80% homologous; however, their expression differs. Vesicle splitting occurs during specialized neuronal function and involves all three dynamins. The pleckstrin homology domain (PH), middle domain (MD), GTPase effector domain (GED), and proline-rich carboxyterminal domain (PR) are the five domains that make up the expanded structure of classical dynamin [191,195,196]. Ras GTPases require guanine exchange factors and GTPase-activating proteins, whereas the GTPase domain is activated by nucleotide-dependent dimerization [188,197]. The GTPase domain contains four GTPase binding motifs: G1, or P-loop, binds phosphate; G2, where threonine coordinates the Mg2+ ion, allows GTP hydrolysis; G3, where aspartate coordinates the Mg2+ ion and glycine binds phosphate; and G4, where nucleotide base is bound [198,199]. Pleckstrin and the PH domain are highly homologous [191]. Three loops show the most variety. Each of them exhibits an area that is primarily hydrophobic and has a positive charge within the binding site to encourage membrane contacts, dynamin polymerization, and membrane curvature [200]. Dynamin’s PH domain binds to phosphatidylinositol-4,5-biphosphate (PI(4,5)P_2_) with a preference over other lipids [201,202]. CME is inhibited by phosphoinositide (PI(4,5)P_2_) binding defects in dynamin mutations with a PH domain [203]. A PRD is unique to classical dynamin. It is a protein–protein interaction domain for many signaling and cytoskeletal proteins, consisting of a number of BAR and SH3 domain binding sites specified by the PXXP motif [186,204]. It functions to bring dynamin to endocytotic locations and coordinate dynamin activity with these other components. The primary role of PRD is to guide dynamin to the cell’s site of action [205,206,207]. In vivo, dynamin I is highly expressed in the central nervous system (CNS), where it mediates synaptic vesicle endocytosis, particularly in response to depolarizing stimulation [208]. Dynamin I has also been found in non-neuronal cell line cultures, even though it typically is not expressed in tissues outside of the CNS [209]. In synapses, dynamin I phosphorylation occurs both at rest and during nerve stimulation. In order to facilitate the interaction of dynamin with endocytic proteins to promote endocytosis, it is quickly dephosphorylated at S774 and S778 by calcium-dependent phosphatase and calcineurin [210]. More specifically, this dephosphorylation is thought to play a role in triggering activity-dependent endocytosis [205]. Dynamin is also brought to the membrane via dephosphorylation, and GTPase activity inhibition may give time for binding to the neck of the developing vesicle before GTPase hydrolyzes to speed up cleavage [191]. All tissues express dynamin II, which plays a significant role in CME. Dynamin II also participates in caveolae budding [211], phagocytosis [212], and cellular functions independent of endocytosis, such as cytokinesis [213] and mitosis [214]. A postsynaptic function of dynamin III is thought to include the formation of specific endocytic sites that recycle AMPA (amino-3-hydroxy-5-methyl-4-isoxazolopropionic acid) receptors found in the protrusions of dendritic spines locally [215].

#### 7.4.1. Long-Chain Amines and Long-Chain Ammonium Salts

**Figure 11:**
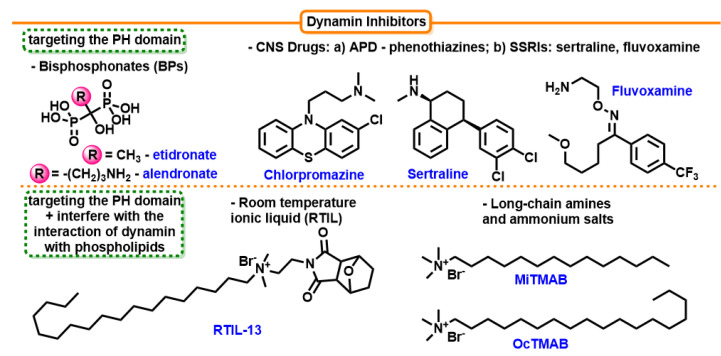
Structures of dynamin inhibitors that affect the pleckstrin homology (PH) domain.

Small-molecule inhibitors, long-chain amines, and long-chain ammonium salts were among the first dynamin and CME inhibitors. The compounds in the series myristyl trimethyl ammonium bromide (MiTMAB) and OcTMAB (Octadecyltrimethylammonium bromide) are surface active and are predicted to alter protein–lipid interactions (Figure 11). MiTMAB is the most active of these compounds, with an IC50 = 3.15 ± 0.64 µM [216]. The suggested mechanism of action of MiTMAB is dynamin–phospholipid inhibition. In non-neuronal cells, MiTMAB inhibits endocytosis of transferrin and EGF on various cell lines. MiTMAB inhibits the GTPase activities of dynamin I or dynamin II. At high concentrations, MiTMAB is a cationic surfactant, as observed with other pharmacologically active cationic amphiphilic compounds, such as chlorpromazine or imipramine [217].

#### 7.4.2. Antipsychotic Drugs (APD)—Phenothiazines

The central ring of phenothiazines contains heteroatoms of nitrogen and sulfur and has a tricyclic heterocyclic structure. APD phenothiazine drugs inhibit GTPases DynI DynII and CME in cells in vitro. It is likely that these compounds work by interfering with the PH domain of dynamin, which is responsible for dynamin detachment. In addition, chlorpromazine did not interfere with AP-2 or clathrin recruitment and did not inhibit CCV formation. These effects are similar for the entire class of APDs. Compounds that inhibited dynamin also showed inhibitory effects on CME. The IC50 of trifluoperazine and its non-APD derivative against DynI was in the region of 2 µM. [218].

#### 7.4.3. Selective Serotonin Reuptake Inhibitors (SSRIs)

Selective serotonin reuptake inhibitors are the common name for a group of drugs that are frequently prescribed as antidepressants to treat major depressive disorder and other mental health issues. In fact, their name originates from the fact that they primarily lead to serotonin reuptake inhibition, with limited effects on noradrenaline, dopamine, and gamma-aminobutyric acid reuptake. SSRIs raise the neurotransmitter’s extracellular level by restricting the neurotransmitter serotonin’s reabsorption into the presynaptic cell. Sertraline and fluvoxamine demonstrated inhibition of DynI with an IC50 of 7.3 ± 1.0 µM and 14.7 ± 1.6 µM, respectively. They target the PH domain of dynamin I, resulting in the inhibition of dynamin-dependent endocytosis [219]. Sertraline acts both on DynI and DynII. Its effect is rapid (detectable after 5 min exposure) and reversible [220].

#### 7.4.4. Room-temperature Ionic Liquid (RTIL)

**Figure 12:**
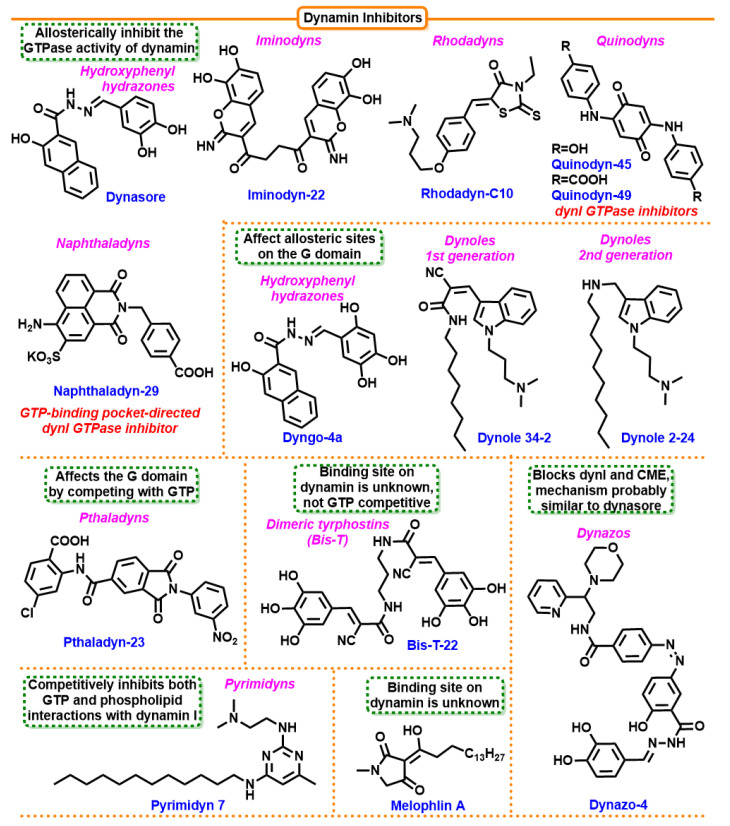
Structures of dynamin inhibitors that inhibit the GTPase activity of dynamin, affect allosteric sites on the G domain, and those with unknown or complex mechanisms of action.

Room-temperature ionic liquid (RTIL) is a substance derived from the Spanish fly aphrodisiac cantharidin. RTILs are imidazole or pyridine salts with a melting point below 150 °C (Figure 12). A group of these compounds showed an inhibitory effect on dynamin by blocking the PH domain. Lengthening the alkyl chain from C4 to C18 increased the inhibitory effect of the compounds. The most active against dynamin GTPase activity was RTIL 13, with 15% inhibition at 300 µM drug concentration and IC50 2.3 ± 0.3 µM [221].

#### 7.4.5. Dimeric Tyrphostins (Bis-T)

Dimeric tyrphostins (Bis-T) exhibit activity against the enzyme dynamin I but have a weak inhibitory effect on CME. The most active dynamin I inhibitor is compound Bis-T-22, with an IC50 of 1.7 ± 0.2 µM. The mechanism of action of this compound is unclear; it neither affects GTP nor competes with GTP or lipids. The inhibitory potency of this class of compounds requires a dimers with two aromatic rings, where the rings contain two -OH groups at the -3,-4 positions; the presence of two free amide -NH or ester -O groups in the linker arm, and the presence of -CN is also of great importance [222]. A new Bis-T-22 prodrug created by adding propionic acid ester has an enhanced ability to pass through membranes; it is rapidly hydrolyzed in the cytoplasm to the parent compound. Despite its strong activity against dynamin, it had a weak effect on CME inhibition. The new prodrug of Bis-T-22 inhibits CME with an IC50 of 8.0 ± 0.5µM [223].

#### 7.4.6. Dynasore

Dynasore was discovered among a library of 16,000 compounds that inhibit CME. It shows activity against the GTPases DynI and DynII, dynamin-related protein 1 (Drp1), and mitochondrial dynamin in vitro (Figure 12). The inhibitory activity of Dynasore is observed 1–2 min after the start of treatment, presumed to be limited by diffusion to the molecular target. This effect is reversible about 20 min after the inhibitor is removed. Dynasore is a non-competitive inhibitor of GTP hydrolysis, with no effect on GTP binding affinity or dynamin self-organization. In addition, testing for DynI inhibition has shown that the compound exhibits inhibitory activity against transferrin endocytosis and the LDL receptor. Unfortunately, this compound has side effects. It binds to serum proteins, which reduces its target inhibitory effect, and to detergents, which may prevent analytic manipulations with this compound in vitro [224].

#### 7.4.7. Dynoles

Dynoles are the first generation of indole-based dynamin inhibitors of DynI GTPase. Dynole 34-2 is the best one with an IC50 of 1.3 ± 0.3 μM (15x more potent than dynasore). This compound is characterized by high lipophilicity and, thus, permeability through cell membranes, which indicates a high level of inhibition of endocytosis. Furthermore, it is not toxic to normal fibroblast cells after exposure to the compound for seven days in culture. The inhibition mechanism involves incompetent binding between dynamin and GTP; dynamin probably binds to the enzyme-substrate complex at a different site than the active site [225].

#### 7.4.8. Iminodines

Iminodines (iminochrome scaffold) are the first nanomolar-range inhibitors of DynI and DynII synthesized on the basis of compounds with the Bis-T pharmacophore. The most potent compounds, Iminodin-17, Iminodin-22, and Iminodin-23, have an IC50 of 330 ± 70 nM, 450 ± 50 nM, and 260 ± 80 nM, respectively. Compared to the earlier class of dynole compounds, new derivatives exhibit five-fold higher activity than Dynole 34-2 or 400-fold that of Dynasore. These compounds incompetently bind the GTPase domain. Iminodyn-22 is the best, exhibiting activity as a broad-spectrum inhibitor against both DynI and DynII [226].

#### 7.4.9. Pthaladyns

Pthaladyns have an inhibitory effect against the GTPase dynamin and SVE (synaptic vesicle endocytosis). These substances are the first class to exhibit competitive inhibitory activity. Pthaladyn-23 was found to be competitive with GTP in cells, and Pthaladyn-29, whose IC50 against dynamins was 4.58 ± 0.06 μM, were the two most promising compounds. With an IC50 of 12.9 ± 5.9 μM, only Pthaladyn-23 proved a potent inhibitor of SVE in brain synaptosomes [227].

#### 7.4.10. Rhodanines

Rhodanine (2-thioxothiazolidin-4-one) skeleton is widely used in medicine, and an attempt was made to use this framework to inhibit CME [228]. Rhodanine derivatives containing N-ethyl (C) and N-acetic acid (E) showed the strongest activity (Rhodadyn-C8 IC50 = 3.0 ± 0.9 µM, Rhodadyn-E9 IC50 = 3.4 ± 0.49 µM), while no inhibition of clathrin was observed at a concentration of 100 µM. Interestingly, not all compounds showed activity against endocytosis inhibition due to insufficient lipophilicity. N-ethyl Rhodadyn-C10 and N-allyl Rhodadyn-D10 were identified as the most potent endocytosis blockers, with IC50 values of 7.0 ± 2.2 and 5.9 ± 1.0 μM, respectively [229].

#### 7.4.11. Dynoles 2

The second generation of indole-based dynamin inhibitors was developed from a series of bisindolylmaleimides (BIMs). They showed a potential inhibitory activity against purified DynI GTPase, with Dynole 2-24 showing the strongest activity with an IC50 of 0.56 ± 0.09 µM. This compound exhibited enhanced DynII inhibition, as well as a slight improvement in DynI vs. DynII selectivity over the first-generation Dynole 34-2. Dynole 2-24 strongly inhibits CME in U2OS cells with an IC50 of 1.9 ± 0.3 µM. In addition, it has a reduced toxicity profile despite the fact that the incorporation of straight alkyl chains can cause off-target membrane activity [230].

#### 7.4.12. Pyrimidines

Pyrimidine derivatives are the first dual-action inhibitors of CME, targeting both dynamin’s interaction with GTP and phospholipids. Pyrimidines also showed effects on CME by examining the uptake of transferrin (Tf) and epidermal growth factor (EGF) in COS7 cells. Both Pyrimidine 6 and 7 at a concentration of 30 µM caused a strong reduction in the internalization of EGF-A488 and Tf-TXR. When evaluated, the IC50 of CME inhibition in non-neuronal cells (by semiautomated CME assay) by the new compounds Pyrimidine 7 in COS-7 cells was 12.1 ± 2.1 µM, and Pyrimidine 6 was 19.6 ± 3.5 µM; similar results were obtained for U2OS cells. The inhibitory effect on CME is reversible 60 min after the removal of the compounds. Incubation of cells with Pyrimidines 6 or 7 (30 µM) disrupted GFP-dynamines I-PH in the plasma membrane—Pyrimidine analogs disrupt the binding of dynamines to the plasma membrane via the PH domain, which is the main mechanism of CME inhibition. Cytotoxicity studies have shown that these compounds do not cause significant damage to cellular lipids but cause growth arrest in 12 cancer cell lines (HT29, SW480—colon carcinoma; MCF-7—breast carcinoma; A2780—ovarian carcinoma; H460—lung carcinoma; A431—skin carcinoma; DU145—prostate carcinoma; BE2-C—neuroblastoma, SJ-G2, U87—glioblastoma; MIA PaCa2—pancreatic carcinoma; SMA (spontaneous murine astrocytoma)—GI50 of about 1 µM at 72 h exposure time [231].

#### 7.4.13. Dyngo

Dyngo is a development of Dynasore with the aim to eliminate the side effects of the latter, such as binding to serum proteins, detergents, and relatively high cytotoxicity. Dyngo group was obtained by condensation of 3-hydroxy-2-naphthoic acid hydrazide with various substituents. Dyngo-4a and Dyngo-6a showed excellent inhibitory activity against DynI (177 and 90 times stronger than the previous generation compound, respectively) both in the presence and in the absence of Tween-80 (T-80) (IC50 2.7 ± 0.7 µM and 0.38 ± 0.05 µM for 4a, and IC50 5.5 ± 0.2 µM and 3.2 ± 0.3 µM for Dyngo-6a, with and without T-80, respectively). In comparison, Dynasore had a much weaker inhibitory effect on DynI in the presence of surfactants (IC50 479 ± 49µM vs. 12.4 ± 1.5 µM in the control). The number and position of hydroxyl groups in the phenyl ring determines both dynamin inhibition activity and detergent sensitivity. Compounds containing at least one -OH at the C3′ or C4′ position are the most sensitive, while removal of this group from C4′ or inclusion at C5′ reduces sensitivity to T-80. Dyngo-6a containing -OH at C2′ and lacking at C4′ is the most detergent-resistant and, at the same time, the most potent DynI inhibitor. The most active compound, Dyngo-4a, inhibits both CME with the IC50 of 5.7 ± 1 µM and SVE at presynaptic nerve terminals. New Dyngo compounds exhibit a preference for inhibiting dynamin in a helical conformation and also bind to detergents stoichiometrically [232].

#### 7.4.14. Naphthaladyn Series

1,8-naphthalamide derivatives were predicted by molecular docking experiments to target the amino-terminal G region of dynamin, which binds and hydrolyzes GTP. Naphtaladyn-23 and Naphthaladyn-29 inhibit DynI activity with the IC50 of 19.1 ± 0.3 µM and 18.5 ± 1.7 µM, respectively. Both compounds inhibit CME with the IC50 of 115 µM and 66 µM, respectively. The lower activity of Naphthaladyn-23 may be due to its lower lipophilicity and, thus, its lower ability to pass through membranes as compared to Naphthaladyn-29 [233].

#### 7.4.15. Quinones

A search in the databases of compounds against DynI activity followed by in silico optimization led to the synthesis of 54 benzoquinone/naphthoquinone-based compounds. Extensive molecular docking analysis suggested several preferential hydrogen bonding and hydrophobic or electrostatic interactions with the binding site. Among these compounds, p-hydroxy and p-carboxy derivatives of aniline called 45 and 49 were the most potent inhibitors of CME with DynI inhibition IC50 of 11.1 ± 3.6 and 10.6 ± 1.6 mM, respectively [234].

#### 7.4.16. Dynazos

Dynazos, the first photosensitive inhibitor of CME, is a derivative of Dynasore with an addition of a photochromic p-azobenzene group. The presence of the azobenzene group does not inhibit the structure’s activity, and Dynazo-3 and Dynazo-4 derivatives inhibit CME in a dose-dependent manner; their activity can be controlled by light [235].

### 7.5. Other Binding Sites for Inhibitors

#### 7.5.1. Nocodazole and Paclitaxel

Paclitaxel, an anticancer drug, targets tubulin, while another anticancer drug, nocodazole, prevents microtubule polymerization. Both paclitaxel, which promotes microtubule assembly, and nocodazole, which promotes microtubule disassembly, alter the dynamics of receptor movement of the endosomal pathway in macropinocytosis. Neither paclitaxel nor nocodazole significantly inhibited endocytosis in the fluid phase. However, paclitaxel caused a redistribution of the fluid phase fluorescent marker to the periphery. Both paclitaxel and nocodazole treatment reduce cargo uptake by 50% after 5 min treatment [236].

#### 7.5.2. Genistein

Genistein is a naturally occurring substance of the isoflavone group that inhibits several tyrosine kinases and SV40-induced caveolin vesicle formation [136]. However, it is not a selective inhibitor of caveolae. For example, Genistein inhibits the uptake by clathrin-coated pits of receptors such as EGF, which require tyrosine phosphorylation for accumulation [237,238]. 

#### 7.5.3. Phenylarsine Oxide

Phenylarsine oxide (PAO) has an arsenic atom in connection with a phenyl group and an oxygen atom. It inhibits both clathrin pathways, macropinocytosis, and phagocytosis. PAO cross-links sulfhydryl groups; therefore, it can inhibit many intracellular targets such as Rho family GTPases [173]. PAO also inhibits oxygen consumption and reduces ATP concentration in cells. The effects of PAO on labeled cargo internalization, ATP content, oxygen consumption, and lactate dehydrogenase (LDH) latency in isolated rat hepatocytes were determined. Treatment with 10 µmol/L PAO for 20 min blocks cargo internalization without affecting ATP concentration [239]. PAO also induces dramatic disorganization of the actin cytoskeleton [204].

## 8. Off-Target Activity of Endocytosis Inhibitors

An important issue with endocytosis inhibitors is their selectivity against a pathway of interest [173]. For example, both Dynasore and Dyngo-4a exert an inhibitory effect on fluid-phase endocytosis in knock-out cells where their target, dynamin, was eliminated, indicating that this activity also represents an off-target effect. In addition, both Dynasore and Dyngo-4a have a strong blocking effect on membrane ruffling [240]. Despite inhibiting clathrin-dependent endocytosis of VEGFR2, Dynasore, Dynole, and Dyngo exert opposite effects on receptor signaling. Dynasore and Dynole have additional activity on VEGFR2 phosphorylation, unlike Dyngo. This suggests additional inhibitory effects on cell signaling beyond the inhibition of endocytosis [241]. Inhibitors of dynamin and endocytosis are also potent suppressors of mTORC1 activation independently of dynamin. Dynasore inhibits RagA by binding to Raptor, reduces mTORC1 recruitment to the lysosome, and inhibits Akt activation and TSC2-S939 phosphorylation, thus reducing mTORC1 activity [242]. Dynasore also reduces cellular cholesterol and disrupts plasma membrane lipid rafts. Due to this ability, it had a protective effect on cells by preserving them from pyolysin from *Trueperella pyogenes* [243]. The absence of absolute specificity does not mean that they cannot be used for research and potential clinical applications. Future research is needed to produce more selective inhibitors of endocytosis.

## 9. Clinical Applications

As endocytosis is linked to several pathological conditions and pathogen entry into cells, it is tempting to block this process. Endocytosis inhibitors have shown promise in clinical trials for various conditions and diseases. They are summarized in Table 2. Probably the most famous was the attempt to use hydroxychloroquine (NCT04437693, NCT04318444, NCT04315896, NCT04318015, NCT04308668), chlorpromazine (NCT04366739), and chloroquine phosphate (NCT04344951) during the COVID-19 epidemics. Although these attempts did not produce the expected results, they demonstrated a good tolerance to these endocytosis blockers. Ruxolitinib and simvastatin have been investigated in a Phase 2 trial (NCT04348695) for their potential to block the entry process used by COVID-19, targeting clathrin-dependent endocytosis. Another Phase 2 trial (NCT04033159) focused on dynamin2 and the antisense oligonucleotide DYN101, which targets dynamin2 pre-mRNA, for the treatment of Centronuclear Myopathy. In a Phase 4 trial (NCT01064284), a chaperone molecule for procoagulant factor VIII (FVIII) was used to block endocytosis in human dendritic cells, thereby protecting FVIII from being endocytosed and subsequently presented to FVIII-specific T cells in patients with Hemophilia A. These clinical trials provide valuable insights into the therapeutic potential of endocytosis inhibitors for various conditions and diseases, including coronavirus infections, Centronuclear Myopathy, Hemophilia A, and pneumonia.

## 10. Questions and Challenges

Endocytosis dysfunctions are observed in many pathological conditions. Thus, endocytosis inhibitors are a promising field for the development of the pharmaceutical industry. Inhibiting endocytosis also inhibits cargo entry into a cell, so one can protect cells from viruses or secreted/circulating proteins, including viral ones. Overcoming resistance and cellular adaptation, which can arise through alternative routes or compensatory mechanisms, is a critical challenge. In these conditions, inhibition of dynamin, which connects most pathways of endocytosis, is probably the best option. Achieving selective targeting and specificity toward desired molecular targets or pathways while minimizing off-target effects is crucial. A rigorous assessment of safety and toxicity is essential to minimize risks. By addressing these challenges, the clinical potential of endocytosis inhibitors can be maximized, paving the way for innovative therapeutic interventions. The field of endocytosis inhibitors holds great promise for the development of novel therapeutics. Addressing the emerging questions and challenges highlighted in this review will provide crucial insights into the optimal design, development, and clinical translation of these inhibitors.

## Figures and Tables

**Table 1:** Pathologies related to endocytosis.

Pathology	Type of Endocytosis	Ligands and Receptors	Molecular Mechanism	References
Cardiac disorders	Caveolin-dependent	eNOS, Cav-1, PCK, L-arginine, L-citrulline, ERK 1/2	After attaching to the CSD (caveolin scaffolding domain), eNOS remains dormant, which lowers NO production. The catalytic domain of eNOS, which is thought to act as the enzyme’s on/off switch, contains a putative binding domain for Cav-1. Cav-1 binding is prevented by mutagenesis in eNOS, although Cav-1’s CSD is less effective at controlling eNOS activity in vivo	[113]
Lipid disorders and atherosclerosis	Clathrin-mediated	RhoA, PKC, LDLR, AP-2, ARH	After binding to the LDL receptor, LDL particles are internalized by clathrin-coated pits into vesicles. Internalization of LDLR requires the presence of AP-2 protein. LDLR interacts with AP-2 indirectly through the adaptor protein ARH. After the fusion of endocytosed vesicles with early endosomes, a drop in pH induces LDL dissociation from its receptor. LDLR is recycled back to the surface of the cell, and LDLs are directed to lysosomes.	[114]
Respiratory diseases	Caveolin-dependent	HSP90, Gq protein, Cav-1, Cav-3, calmodulin, L-arginine, NO	Caveolins participate in Ca^2+^ handling in airway smooth muscle. Caveolins probably promote smooth muscle contraction by regulating Gq protein function and phosphoinositide metabolism. In addition, recruitment across the plasma membrane of RhoA and PKC is dependent on Cav-1. Negative allosteric modulation of eNOS by Cav-1 and 3 competes with positive allosteric modulation of eNOS by Ca^2+^/calmodulin and HSP90 complexes. Thus, in the presence of elevated levels of Ca^2+^/calmodulin and HSP90, the inhibitory effect of Cav-1 on NOS activity can be completely reversed.	[115,116,117,118]
Cancer	Clathrin-mediated	Cdc42, Cbl, b-Pix EGFR, Rac1, Src, RhoA	Cdc42 bound to GTP forms a complex mediated by the adaptor protein b-Pix and Cbl. As a consequence, Cbl cannot react with EGFR; this promotes ubiquitination and degradation of the receptor. Cdc42 belongs to the small GTPase family, which represents critical effectors of pathways and Ras-derived signaling that have a proven role in tumor cell invasiveness and metastasis.	[119]

**Table 2:** Clinical trials of endocytosis modulators.

Target	Compound	Condition or Disease	Mechanism	Number	Status	References
Blocking clathrin-dependent endocytosis	Ruxolitinib and simvastatin	Coronavirus Infection	Block the entry process used by COVID-19	NCT04348695	Phase 2	[244,245]
Dynamin2	DYN101	Centronuclear Myopathy	Antisense oligonucleotide directed against dynamin2 pre-mRNA	NCT04033159	Phase 2	[246]
Blocking endocytosis in human dendritic cells	Chaperone molecule for procoagulant factor VIII (FVIII)	Hemophilia A	VWF protects FVIII from being endocytosed by human dendritic cells and subsequently presented to FVIII-specific T cells	NCT01064284	Phase 4	[247,248]
Reducing the expression of phosphatidylinositol-binding clathrin assembly protein and blocking the clathrin-mediated endocytosis	Hydroxychloroquine (HCQ)	Coronavirus Infection (COVID-19)	Inhibits the ability of viruses to escape into the host cell and start replicating	NCT04437693	Phase 3	[249]
		Coronavirus Infection (COVID-19)		NCT04318444	Phase 3	[250,251]
		COVID-19, Severe Acute Respiratory Syndrome		NCT04315896	Phase 3	[252]
		COVID-19, Severe Acute Respiratory Syndrome		NCT04318015	Phase 3	[253]
		Coronavirus Infection (COVID-19)		NCT04308668	Phase 3	[254]
		Coronavirus Infection (COVID-19)		NCT04316377	Phase 4	[255,256]
Blocking clathrin-mediated endocytosis	Chlorpromazine (CPZ)	Coronavirus Infection (COVID-19)	CPZ affects the translocation of the clathrin and AP2 from the cell surface to intracellular endosomes	NCT04366739	Phase 3	[257]
Inhibiting endosome acidification	UNIKINON (Chloroquine phosphate)	Pneumonia, Coronavirus Infection (COVID-19)	Blocks the endosomal-mediated viral entry	NCT04344951	Phase 2	[258]

## Data Availability

No new data were created.
